# Nutrition Label Utilization, Dietary Self-Management, and Health-Related Quality of Life Among Korean Adults: A Two-Part Model Analysis of Nationally Representative Survey Data

**DOI:** 10.3390/healthcare14101419

**Published:** 2026-05-21

**Authors:** Yoonjin Lee

**Affiliations:** Health Institute of Healthy Aging Society, Konkuk University, Seoul 05029, Republic of Korea; leeyoonjin@konkuk.ac.kr; Tel.: +82-10-3077-9260

**Keywords:** health-related quality of life, EQ-5D, nutrition label, dietary self-management, two-part model, KNHANES, Korea, social determinants of health

## Abstract

**Background:** Health-related quality of life (HRQoL) is a central outcome measure in population health research, yet empirical investigations directly linking nutrition label utilization to HRQoL remain limited, particularly in East Asian contexts. This study examines the associations between nutrition label use, dietary control, and HRQoL among Korean adults while accounting for the pronounced ceiling effect inherent in EQ-5D utility scores. **Methods:** Data were drawn from the Korea National Health and Nutrition Examination Survey (KNHANES) 2024 (N = 5215 adults aged 19–80). HRQoL was measured using the EQ-5D-3L with Korean time trade-off weights. Nutrition label use was operationalized as a composite index (0–3). Given that 48.0% of the weighted sample reported perfect health, a two-part model was employed: Part 1 applied survey-weighted logistic regression predicting perfect health, while Part 2 applied survey-weighted OLS regression restricted to those with imperfect health (n = 2713). **Results:** In Part 1, nutrition label use was not significantly associated with perfect health (OR = 1.057, *p* = 0.124), whereas dietary control was negatively associated (OR = 0.819, *p* = 0.009), suggesting reverse causality. In Part 2, nutrition label use was positively associated with EQ-5D scores (β = 0.0047, *p* = 0.006). Education, income, and unmet medical need were dominant predictors. Results were robust to an alternative full-sample OLS specification. Conclusions: Nutrition label utilization was modestly and positively associated with HRQoL among Korean adults with imperfect health. Given the cross-sectional design, this association should be interpreted as exploratory and may reflect broader health-oriented characteristics, including health consciousness, self-regulatory behaviors, and health literacy, rather than the independent effect of nutrition label use alone. The findings also underscore the methodological importance of addressing ceiling effects in EQ-5D analyses.

## 1. Introduction

Health-related quality of life (HRQoL) is a widely used composite indicator in health research, policy, and clinical practice that captures the subjective experience of health across multiple domains—physical functioning, pain, psychological well-being, and social participation [1,2]. As non-communicable diseases have become the dominant burden on global health systems, preference-based HRQoL instruments are now widely used in cost-effectiveness analyses, population surveillance, and policy planning [3,4]. In Korea, rapid epidemiological and nutritional transitions over the past three decades have raised the prevalence of obesity, metabolic syndrome, and associated comorbidities, motivating attention to the behavioral and structural correlates of population HRQoL.

EQ-5D-3L [4] is a widely used generic preference-based HRQoL instrument that scores five health dimensions onto a single utility index. Korean-specific time trade-off weights [5,6] enable nationally appropriate scoring; detailed operationalization is provided in Section 3.2.1.

Diet quality and nutritional behavior are major modifiable correlates of chronic disease risk and, by extension, of HRQoL [2,7]. Within this framework, nutrition label use on packaged foods is a proximal behavioral correlate of diet literacy that has been associated with healthier purchasing and consumption decisions in observational research [8,9]. Individuals who consult nutrition labels at the point of purchase are more likely to select lower-calorie, lower-sodium, and nutritionally superior products, and label-driven product selection has been correlated with improved dietary quality and, in cross-sectional studies, with more favorable downstream health indicators [10,11].

In Korea, mandatory back-of-pack nutritional information has been required for most processed foods since 2010 [12], with a sodium comparative display added in 2016. Unlike Chile, Australia/New Zealand, and France—which have adopted warning-label, Health Star Rating, or Nutri-Score systems—Korea has not adopted a mandatory front-of-pack (FOP) interpretive labeling regime. Because back-of-pack numerical labels are less effective at shaping consumer behavior than interpretive FOP systems [13,14,15], the Korean case is informative: any observed association between label use and health outcomes arises under a regime that demands active consumer effort to locate and interpret information.

Despite the theoretical plausibility of a label-use → HRQoL pathway, direct empirical evidence remains sparse. Most prior studies have examined either the antecedents of label use or its proximal influence on dietary choices, without extending the outcome chain to broader utility measures [8,9,13]. The cross-sectional relationship between dietary self-management and HRQoL is further complicated by reverse causality, since individuals with lower HRQoL may report more frequent dietary control. Compounding these substantive challenges is a methodological one: the pronounced EQ-5D ceiling effect—a large fraction of community-dwelling adults reports perfect health—violates the assumptions of standard regression approaches.

Established health-behavior theory clarifies why nutrition labels would matter for HRQoL beyond the mere availability of information. Within the Health Belief Model [16], label awareness corresponds to perceived susceptibility and severity, whereas active label use and label-driven purchasing reflect perceived benefits and self-efficacy in mitigating diet-related risk. Social Cognitive Theory [17] further locates label-driven dietary choice within reciprocal interactions among personal capability, behavioral self-regulation, and environmental supports—of which the labeling regime is part. Through this lens, the cumulative engagement stages captured by the present index—awareness, use, and purchase influence—represent successive expressions of health literacy [18], information-processing behavior [10,11], and preventive health orientation, providing a behavioral-mechanism rationale for examining their cumulative association with HRQoL.

**Figure 1:**
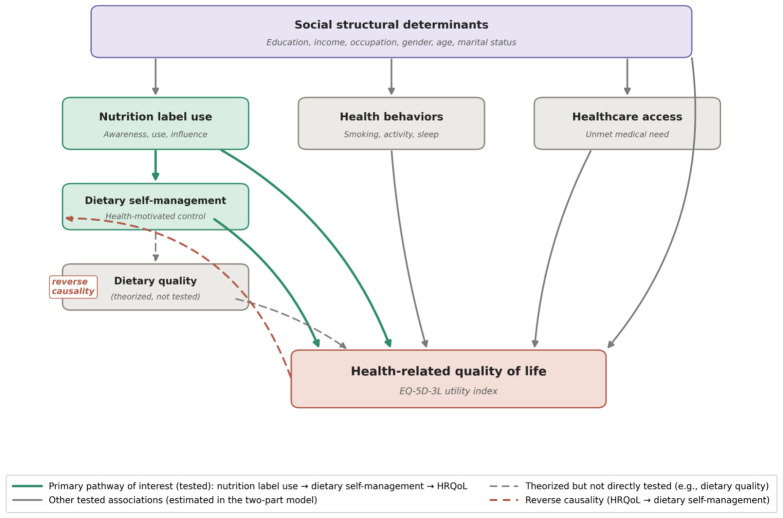
Conceptual framework.

This study addresses these gaps by analyzing data from the Korea National Health and Nutrition Examination Survey (KNHANES) 2024, the most recent nationally representative health and nutrition survey of the Korean civilian population. The study employs a two-part model incorporating complex survey design adjustments to examine: (1) the sociodemographic and behavioral predictors of the probability of reporting perfect HRQoL, and (2) the correlates of HRQoL utility scores among adults with imperfect health, with a particular focus on the roles of nutrition label use and dietary self-management. Figure 1 presents the conceptual framework guiding this investigation, illustrating the hypothesized associations among social structural determinants, nutrition label engagement, dietary self-management, health behaviors, healthcare access, and HRQoL, including the possible reverse-causality pathway from health impairment to dietary control. This framework should be interpreted as a conceptual guide for testing cross-sectional associations rather than as a causal pathway model.

Figure 1 represents the conceptual framework for the associations among social structural determinants, nutrition label use, dietary self-management, health behaviors, healthcare access, and health-related quality of life (HRQoL). Solid arrows represent associations directly estimated in this study. Dashed gray arrows represent theorized but not directly tested mediating associations (e.g., dietary quality). The dashed red arrow represents the possible reverse-causality pathway from health impairment to dietary self-management. Of the depicted pathways, only the solid-line associations from social structural determinants, nutrition label engagement, dietary self-management, health behaviors, and healthcare access to HRQoL are directly estimated within the two-part model reported in this study; mediating pathways through dietary quality, nutritional status, and chronic-disease burden are theorized on the basis of prior evidence but not empirically tested in the present analysis. Therefore, the figure should be read as a conceptual framework for cross-sectional association testing rather than as a causal model.

## 2. Literature Review

### 2.1. Nutrition Label Utilization and Health Outcomes

The accumulating evidence on nutrition labeling has yielded a more qualified view of label efficacy than earlier optimism suggested, and three lines of tension structure the contemporary literature.

The first concerns format heterogeneity. Cowburn and Stockley’s [8] foundational review documented that consumer attention does not translate uniformly into label use, with comprehension varying by educational background and health orientation; subsequent work by Campos et al. [9] linked label use to healthier food choices and lower energy intake. The magnitude of these effects has proven sensitive to interpretive design, however, and recent syntheses have repeatedly drawn this distinction sharper. The interdisciplinary meta-analysis by Ikonen et al. [13], synthesizing 114 studies of front-of-package labeling, found that interpretive aids helped consumers identify healthier products yet exerted limited and sometimes halo-prone effects on actual purchasing. An et al. [14] further differentiated format effects, reporting that traffic-light and warning-label systems generated more consistent purchase-behavior shifts than health-star or daily-intake formats—a pattern subsequently corroborated by the Cochrane-method synthesis of Kelly et al. [19], which assigned moderate-certainty status to the proposition that front-of-package labeling improves consumer understanding and choice while encouraging favorable industry reformulation. Taken together, this body of work argues against treating “labeling” as a unitary intervention: the empirical record favors interpretive over numerical formats, and warning-label or traffic-light systems over star ratings or daily intake guides.

The second concerns the equity dimension. Shrestha et al. [15] documented that socioeconomic position moderates label efficacy, with lower-SES consumers showing smaller behavioral shifts—a finding with direct implications for whether labeling policies redistribute or entrench dietary inequalities. This moderation has rarely been engaged in the policy literature on Korean labeling regimes, despite its evident relevance to a context in which the educational gradient in label use is itself steep [20].

The third tension, and the one most consequential for the present study, concerns the outcome chain. The cumulative evidence base traces label engagement through to dietary choice and, in selected cases, to nutritional intake or industry reformulation, but rarely extends to multidimensional preference-based outcomes such as EQ-5D. The implicit public-health rationale for labeling policy—that better-informed choices aggregate over time into measurable population-health gains—therefore rests on extrapolation rather than on direct evidence linking label engagement to generic health utility. The present study addresses this evidentiary gap by examining whether nutrition label utilization is cross-sectionally associated with EQ-5D utility scores in a nationally representative Korean adult sample.

### 2.2. Dietary Self-Management and the Challenge of Reverse Causality

Dietary self-management—the deliberate modification of food intake to address health conditions or achieve health goals—is a cornerstone of chronic disease management [2]. Yet, the cross-sectional relationship between dietary control and HRQoL is complicated by reverse causality: individuals already experiencing health impairments are more likely to have been advised or motivated to engage in dietary modification. In cross-sectional data, this generates a paradoxical negative association between dietary control and HRQoL—not because dietary control harms health, but because those with poorer health are more likely to engage in it. The present study explicitly models this interpretive challenge within the two-part model framework, treating the dietary control coefficient as indicative of the direction of association while acknowledging the limitations of causal inference from cross-sectional data.

### 2.3. Ceiling Effects in EQ-5D and Methodological Approaches

A frequently neglected methodological issue in HRQoL research is the pronounced EQ-5D ceiling effect: a substantial fraction of community-dwelling adults reports perfect health, producing a point mass at the upper bound. The Cheng et al. [20] meta-analysis (94 studies, 37 countries, >4.5 million adults) reports global pooled ceiling shares of 56% (3L) and 49% (5L), with East and Southeast Asian populations being notably higher. The present sample’s 48% ceiling share is consistent with these benchmarks and with prior KNHANES analyses [21,22].

Applying standard ordinary least squares (OLS) regression to such data conflates two qualitatively distinct processes: the determinants of achieving versus not achieving perfect health, and the determinants of the level of health utility among those who fall below perfect health. The two-part model (TPM), originally developed in health econometrics by Duan and colleagues [23] and formalized by Mullahy [24], addresses this distributional complexity by separately modeling these two processes. Comparative methodological research has demonstrated that the TPM outperforms standard OLS, Tobit, censored least absolute deviations (CLADs), and beta regression models under conditions of heavy ceiling effects in EQ-5D data [25]. The present study employs a TPM (logistic-OLS) approach, providing both the probability of perfect health (Part 1) and the conditional distribution of utility scores among those with imperfect health (Part 2).

### 2.4. Social Determinants and Health Behaviors as Predictors of HRQoL

The social determinants of health framework posits that health outcomes are patterned by social position across the life course [26,27]. In Korea, Nguyen et al. [28] reported that adults with chronic disease in higher income brackets had a 1.95-fold higher likelihood of better HRQoL, and Kim et al. [22] documented persistent sociodemographic gradients in EQ-5D-3L across genders, age groups, and income levels in 2.8 million Korean adults (2008–2021). Gender differences are consistently documented internationally, with women systematically reporting lower HRQoL than men [21,29], and aging is robustly negatively associated with HRQoL through accumulated chronic disease and functional decline [1].

Among modifiable health behaviors, smoking [30], sedentary behavior [31], insufficient physical activity, and short or long sleep duration are robustly associated with lower HRQoL [32]; the alcohol–HRQoL relationship is contested and may reflect abstainer heterogeneity rather than a genuine protective effect [7]. Unmet medical need—the self-reported failure to obtain needed care—is a central indicator of healthcare access [33] with direct HRQoL implications, and even within Korea’s near-universal insurance system, geographic, financial, and literacy barriers can prevent timely utilization.

The present study situates nutrition label use and dietary self-management within this comprehensive framework of social determinants and health behaviors, controlling for gender, age, education, income, occupational class, marital status, living arrangement, smoking, drinking frequency, sedentary time, walking frequency, sleep duration, and unmet medical need.

## 3. Data and Methods

### 3.1. Data Source

This study utilized data from the Korea National Health and Nutrition Examination Survey (KNHANES) 2024, the third period of the ninth cycle of this nationally representative cross-sectional survey. KNHANES has been conducted annually since 1998 by the Korea Disease Control and Prevention Agency (KDCA) as the primary national surveillance system for population health status, health behaviors, and nutritional intake in the Republic of Korea [12]. The survey encompasses three integrated components: a Health Interview Survey collecting information on self-reported health status, chronic disease diagnoses, healthcare utilization, and health behaviors; a Health Examination Survey conducting standardized physical and biochemical measurements; and a Nutrition Survey assessing dietary intake through 24 h dietary recall and food frequency questionnaires.

KNHANES employs stratified multistage cluster probability sampling from the national resident registration database as the sampling frame. In the first stage, primary sampling units (PSUs) are geographically defined clusters of households stratified by administrative region (si/do) and urbanicity. In the second stage, households are randomly selected within PSUs. All eligible household members aged one year and above are invited to participate. Data collection is conducted through household visits by trained interviewers and medical staff from mobile examination centers, ensuring standardized administration across diverse geographic settings. Survey procedures have been reviewed and approved by the Institutional Review Board of the Korea Disease Control and Prevention Agency, and all participants provided written informed consent.

The KNHANES 2024 achieved a total sample of N = 6997 respondents across all ages. The present analysis was restricted to adults aged 19 to 80 years with complete data on all analytical variables (N = 5215 after listwise deletion). From the 6997 source respondents, 1015 fell outside the 19–80 age-eligibility criterion and a further 767 were excluded by listwise deletion of cases with item non-response on nutrition-label-use items, dietary control, and sedentary time. Sensitivity analyses comparing included and excluded cases on key observed characteristics—including age, gender, educational attainment, income quintile, and EQ-5D utility scores—confirmed that listwise deletion did not introduce substantive systematic bias in the distribution of the primary outcome variable, though excluded cases tended to be older and less educated. The possibility of residual selection bias from non-random missingness is acknowledged as a limitation in the Discussion.

**Figure 2:**
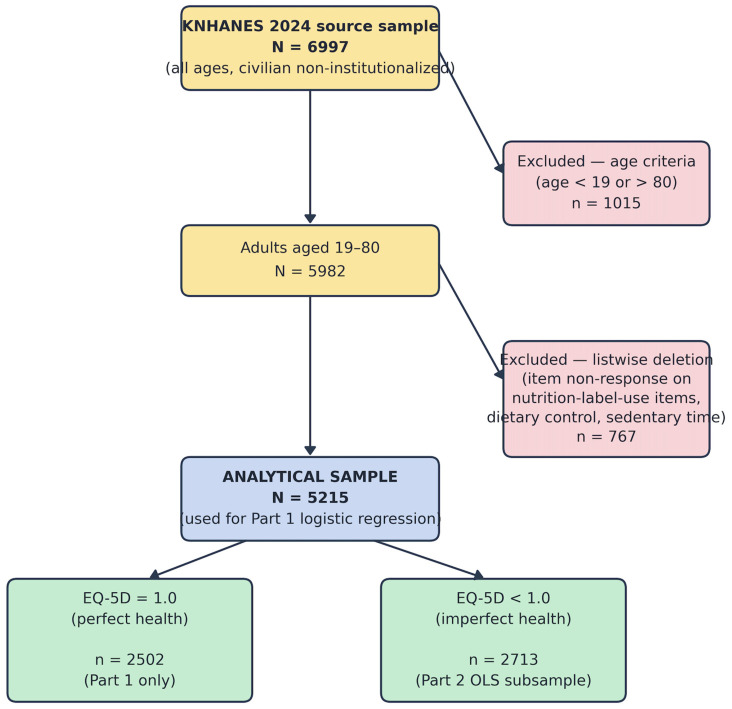
Participant-flow diagram. showing the derivation of the analytical sample (N = 5215) from the KNHANES 2024 source sample (N = 6997). The Part-2 OLS subsample (n = 2713) corresponds to respondents with EQ-5D utility scores below the ceiling of 1.0.

Figure 2 presents the participant-flow diagram from the KNHANES 2024 source sample to the analytical sample reported here, following the structure recommended for cross-sectional observational studies. The 1015 respondents excluded on age criteria comprised participants younger than 19 or older than 80 years, who fall outside the population to which the substantive research question applies. The 767 respondents excluded by listwise deletion were those with item non-response on the analytical variables, concentrated on the three nutrition-label-use items, dietary control, and sedentary time. The full analytical sample of N = 5215 was retained for Part 1 of the two-part model, and the imperfect-health subsample of n = 2713 (those with EQ-5D < 1.0) was used for Part 2.

### 3.2. Variables and Measurement

#### 3.2.1. Dependent Variable: Health-Related Quality of Life

HRQoL was measured using the EQ-5D-3L, a generic preference-based health utility instrument comprising five dimensions—mobility, self-care, usual activities, pain/discomfort, and anxiety/depression—each assessed at three severity levels (no problems, some problems, extreme problems/unable). Each unique combination of dimension-level responses constitutes a health state. Utility scores were calculated by applying the Korean TTO value set developed by Lee and colleagues [5], which was derived from a representative Korean general population sample using the time trade-off method. The resulting utility index is anchored at 1.0 (full health) and 0.0 (a health state equivalent to death), with negative values possible for states considered worse than death; the empirical range in the analytical sample was −0.013 to 1.000 (M = 0.928, SD = 0.096).

#### 3.2.2. Main Independent Variables

Nutrition label use index was constructed as a composite of three binary KNHANES items assessing (1) awareness of nutrition labels on processed food packaging (LK_LB_CO: “Have you noticed nutrition labels on processed food products?”), (2) active use of nutrition labels when purchasing processed foods (LK_LB_US: “Have you checked nutrition labels when purchasing processed foods in the past year?”), and (3) purchase behavior influenced by nutrition label information (LK_LB_EF: “Has nutrition label information influenced your food purchasing decisions?”). Each affirmative response was coded 1 and negative response coded 0, with the three items summed to yield an index ranging from 0 (no awareness, use, or influence) to 3 (all three components present; M = 1.591, SD = 1.081).

The nutrition label use index is conceptualized as a formative composite rather than a reflective scale. The three items capture conceptually distinct and hierarchically ordered stages of label engagement—awareness, behavioral use, and purchase influence—that represent a cumulative progression of engagement depth rather than interchangeable indicators of a single latent construct [10]. In formative measurement, internal consistency (e.g., Cronbach’s alpha) is not a necessary validity requirement because the items are causes rather than effects of the composite score; an individual may be aware of labels without using them, and may use them without being influenced in purchasing decisions [34]. The additive index thus represents a cumulative count of engagement stages, with higher values indicating more complete integration of nutrition label information into food purchasing behavior. This formative operationalization is consistent with how nutrition label engagement has been measured in prior population survey research [8,10] and with established methodological guidance on index construction with formative indicators [34].

Because KNHANES 2024 administers the three nutrition-label items under a conditional skip pattern—the use question is asked only of respondents reporting awareness, and the influence question only of respondents reporting use—the four observed values of the index correspond to mutually exclusive cumulative stages of label engagement: not aware (0), aware but not using (1), using without purchase influence (2), and using with purchase influence (3). The composite is therefore most accurately interpreted as a count of cumulative engagement stages rather than a free additive combination of three independent binary indicators, an interpretation consistent with the formative-measurement framing above. The empirical adequacy of treating these four values as a continuous score is examined through the component-decomposition and ordinal-treatment sensitivity analyses reported in Section 4.4.1 and Section 4.4.2.

Dietary control was assessed through the KNHANES health interview item (N_DIET): “Are you currently controlling your diet for reasons of health, weight management, or disease management?” Responses were dichotomized as yes (1) versus no (0), with 32.6% of the weighted sample reporting current dietary control. This item captures intentional, health-motivated dietary modification but does not distinguish specific dietary strategies or the duration of dietary control, a limitation acknowledged in the Section 5.

#### 3.2.3. Control Variables

Sociodemographic controls included: gender (male = reference, female = 1); age in continuous years; educational attainment (four categories: elementary school or below = reference, middle school, high school, college or above); household income quintile (Q1 = lowest = reference, through Q5 = highest), calculated from equivalized household income; occupational class (seven categories: manager/professional = reference, clerical, service/sales, agriculture, skilled labor, elementary labor, unemployed/inactive), classified according to the Korean Standard Classification of Occupations (KSCO); marital status (married = reference, unmarried including never married, divorced, widowed); and living arrangement (living alone = reference, couple only, couple with children, other household types).

Health behavior controls included: smoking status (non-smoker = reference, current smoker); drinking frequency (0 = non-drinker to 6 = daily), from a seven-point ordinal scale; daily sedentary time in hours; weekly walking days (0–7); and average daily sleep duration in hours, computed as (weekday sleep hours × 5 + weekend sleep hours × 2)/7, derived from bedtime and wake-up time items. Unmet medical need was assessed by the item “In the past 12 months, was there a time when you needed medical care but did not receive it?” (yes = 1, no = 0).

Table 1 below summarizes, for each construct entered into the two-part model, the conceptual definition motivating its inclusion, the operational measurement employed in KNHANES 2024, and the methodological justification for the chosen operationalization. Variables introduced solely for sensitivity-analysis purposes—chronic-disease composite and PHQ-9 depression-screening score, both discussed in Section 5.3 as residual-confounding considerations—are listed at the bottom of the table for completeness.

### 3.3. Analytical Strategy

All analyses incorporated the complex survey design of KNHANES 2024 using a design object specified with stratification variable kstrata, primary sampling unit variable psu, and integrated sampling weight wt_tot that combines the health interview, health examination, and nutrition survey weights to represent the Korean civilian adult population. Failure to account for the stratified cluster design would yield biased standard errors and incorrect inferential statistics [12].

The two-part model was selected as the primary analytical framework to address the pronounced ceiling effect in EQ-5D utility scores. When 48.0% of the weighted sample reports perfect health, the standard OLS regression model is inappropriate because the outcome distribution is a mixture of a point mass at 1.0 and a continuous distribution on the open interval. The two-part model decomposes the outcome into two processes: a binary indicator of whether the outcome equals its maximum value (Part 1), and the conditional distribution given that it is below the maximum (Part 2) [23,24]. The TPM was preferred over alternative approaches—including Tobit, CLAD, and beta regression—for three methodological reasons: (a) the Tobit model assumes a latent variable exceeding the observed upper bound, which lacks theoretical justification when perfect health is a genuine, observed state rather than a censored observation; (b) CLAD, while robust to non-normality, has been shown to perform poorly relative to TPM in predictive accuracy under heavy ceiling effects [25]; (c) beta regression requires transformation of the bounded outcome and cannot accommodate the point mass at 1.0. Simulation studies have confirmed that the TPM yields superior predictive accuracy under the distributional characteristics typical of EQ-5D data in community-dwelling populations [20,25].

Part 1 employed survey-weighted logistic regression with the binary-dependent variable defined as EQ-5D = 1.0 (perfect health = 1) versus EQ-5D < 1.0 (imperfect health = 0). The quasibinomial family was specified to account for potential overdispersion in survey-weighted models. Results are reported as odds ratios (ORs) with 95% confidence intervals and Wald test *p*-values. Part 2 employed survey-weighted ordinary least squares regression restricted to the subsample with EQ-5D < 1.0 (n = 2713 unweighted observations), with EQ-5D utility score as the continuous dependent variable. Results are reported as unstandardized beta coefficients with 95% confidence intervals and *t*-test *p*-values.

Robustness was assessed through an alternative OLS model estimated on the full analytical sample (N = 5215) with survey weights. Multicollinearity was assessed using variance inflation factors (VIF) and generalized VIF (GVIF) for multi-category variables; all values were below the conventional threshold of 5, with the highest GVIF^(1/2Df) value of 1.737 observed for age. All analyses were conducted in R version 4.3.1 using the survey package. This study is reported in accordance with the Strengthening the Reporting of Observational Studies in Epidemiology (STROBE) guidelines for cross-sectional studies [35].

Several additional sensitivity analyses were conducted to assess the robustness of the main findings and are reported in Section 4.4. These comprise: (i) Part-2 residual diagnostics—Shapiro–Wilk normality and Breusch–Pagan heteroscedasticity tests—together with HC3 heteroscedasticity-consistent standard errors estimated on the unweighted Part-2 OLS specification; (ii) decomposed and ordinal specifications of the nutrition-label-use index, including a polynomial-trend test of the linear functional form; (iii) multiple-imputation (MICE, m = 10) re-analysis to address the listwise-deletion exclusion of 25.5% of the original sample; (iv) a dietary-control × label-use interaction model with stratified estimates; (v) an alternative full-sample OLS specification reported in Section 4.3. All sensitivity analyses retained the KNHANES complex survey design (kstrata, psu, wt_tot) and used the same control-variable set as the main two-part model. The two-part model is presented as one defensible analytical strategy for bounded outcomes with substantial ceiling effects rather than as the only such strategy, and the alternative modeling families noted above (Tobit, CLAD, fractional logit, and beta regression on a transformed disutility) represent a useful empirical-comparison target for future re-analyses.

## 4. Results

### 4.1. Descriptive Statistics

Table 2 presents the weighted sample characteristics for the analytical sample (N = 5215). The mean age was 49.66 years (SD = 16.94), and 50.3% were female. Nearly half (47.1%) had college-or-above education. The mean EQ-5D utility index was 0.928 (SD = 0.096), with 48.0% of the weighted sample at the ceiling (EQ-5D = 1.0). The mean nutrition label use index was 1.591 (SD = 1.081), indicating moderate average engagement with nutritional labeling.

Table 3 reports the empirical range of the principal continuous variables to complement the means and SDs reported in Table 2.

**Figure 3:**
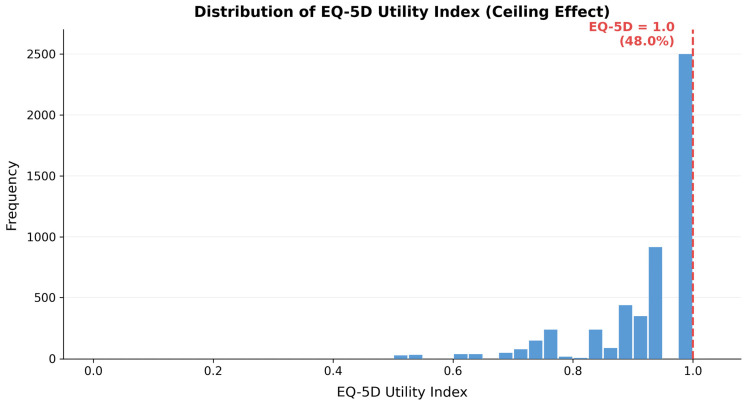
Distribution of EQ-5D Utility Index. Note: The dashed vertical line indicates EQ-5D = 1.0 (48.0% of the weighted sample).

Figure 3 presents the distribution of the EQ-5D utility index. The ceiling at 1.0 accounted for 48.0% of the weighted sample, and the remaining distribution was left-skewed with a minimum of −0.013. The combination of a point mass at the upper bound and a continuous left tail informed the choice of a two-part model.

Table 4 presents Pearson correlations among the primary study variables. The strongest bivariate predictor of EQ-5D was education (r = 0.300), followed by age (r = −0.270), drinking frequency (r = 0.175), nutrition label use index (r = 0.166), and unmet medical need (r = −0.161). Notably, nutrition label use was itself strongly patterned by social position: higher education (r = 0.412) and younger age (r = −0.410) were associated with greater label use, underscoring the importance of controlling for sociodemographic characteristics when estimating the net effect of nutrition label use on HRQoL.

**Figure 4:**
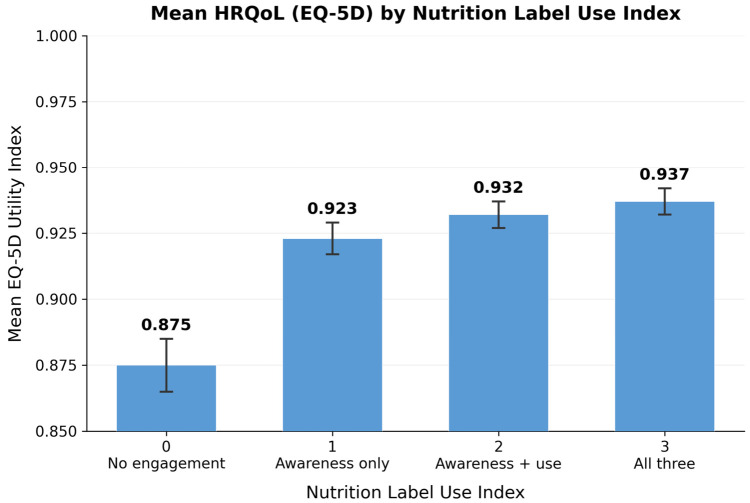
Mean HRQoL. Note: The index sums three binary components: awareness of nutrition labels, use of labels when selecting food, and influence of label information on purchase decisions (0 = none; 3 = all three). Error bars represent 95% confidence intervals under the complex survey design.

Figure 4 presents the weighted mean EQ-5D utility index by nutrition label use index. Mean utility rose monotonically across the four levels: 0.875 at index = 0, 0.923 at index = 1, 0.932 at index = 2, and 0.937 at index = 3. The largest single-step increment occurred between no engagement and awareness-only (+0.048), with smaller increments thereafter (+0.009 and +0.005).

### 4.2. Two-Part Model Results

#### 4.2.1. Part 1: Logistic Regression Predicting Perfect Health (EQ-5D = 1.0)

Table 5 presents the results of the survey-weighted logistic regression predicting the probability of reporting perfect health (EQ-5D = 1.0). Nutrition label use index was not significantly associated with the odds of perfect health (OR = 1.057, 95% CI [0.985, 1.134], *p* = 0.124), suggesting that among those in the healthy portion of the population, incremental variation in label use does not further differentiate the probability of perfect versus imperfect health. Active dietary control was significantly and negatively associated with perfect health (OR = 0.819, 95% CI [0.705, 0.951], *p* = 0.009), providing clear evidence consistent with reverse causality: individuals with health conditions that necessitate dietary management are less likely to report perfect health, not because dietary control itself is harmful but because the direction of association runs from impaired health to dietary modification.

Sociodemographic and behavioral correlates patterned the probability of reporting perfect health in expected directions (Table 5): female gender, advancing age, current smoking, and longer daily sedentary time were each significantly negatively associated with the odds of perfect health, while college-or-above education was the only educational contrast reaching significance. Income quintiles showed no significant Part-1 gradient, suggesting that income effects on HRQoL emerge more strongly in differentiating utility levels within the imperfect-health range than in determining entry into the perfect-health state. Unmet medical need was the largest single Part-1 predictor (OR = 0.315, 95% CI [0.248, 0.401], *p* < 0.001), reducing the odds of reporting perfect health by approximately two-thirds—a magnitude consistent with the interpretation of access barriers as both a marker of underlying disease burden and an independent contributor to functional impairment.

**Figure 5:**
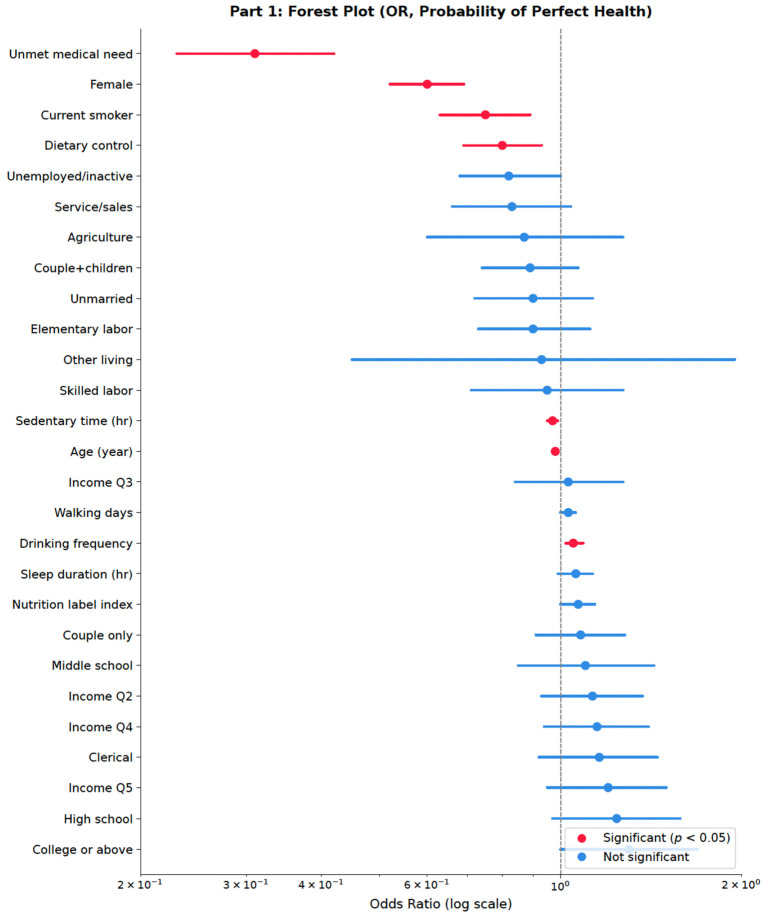
Forest Plot(1). Note: Figure 5 from the Part 1 survey-weighted logistic regression predicting perfect health (EQ-5D = 1.0 vs. <1.0; N = 5215). Red markers indicate *p* < 0.05; blue markers indicate non-significance. The vertical dashed line at OR = 1.0 represents the null. The x-axis is on a log scale. Reference categories: elementary or below (education); Q1 (income); manager/professional (occupation); living alone (living arrangement).

Figure 5 presents the Part 1 results in forest plot form. Unmet medical need (OR = 0.315) was the largest predictor of reduced odds of perfect health, followed by female sex (OR = 0.584), current smoking, and active dietary control. The 95% confidence interval for the nutrition label use index spanned OR = 1.0, consistent with the non-significant Part 1 association reported in Table 5.

Drinking frequency was the only behavioral covariate with a significant positive association with perfect health in Part 1 (OR = [1.047]), a pattern that likely reflects reverse selection—individuals with chronic disease or dietary management restrictions reduce alcohol intake.

#### 4.2.2. Part 2: OLS Regression Predicting EQ-5D Among Those with Imperfect Health

Table 6 presents the results of the survey-weighted OLS regression predicting EQ-5D utility scores among the subsample reporting imperfect health (EQ-5D < 1.0; n = 2713). The model explained 18.0% of the variance (R^2^ = 0.180).

Nutrition label use index was positively and significantly associated with EQ-5D (β = 0.0047, 95% CI [0.0013, 0.0081], *p* = 0.006), such that each unit increase in the index was associated with a 0.0047-unit increase in EQ-5D utility after comprehensive adjustment for sociodemographic characteristics, health behaviors, and healthcare access.

Dietary control was again negatively associated (β = −0.0091, *p* = 0.016), consistent with the reverse causality interpretation. Sociodemographic position structured Part-2 EQ-5D in the directions anticipated under the social-determinants framework (Table 6): female gender, advancing age, unmarried status, current smoking, and longer daily sedentary time each carried significant negative associations, and a clear monotonic gradient was observed across both education—with all three non-reference categories significantly higher than the elementary-or-below reference—and household income quintiles, with all four upper quintiles significantly higher than Q1. Among occupational categories, agricultural workers and the unemployed/economically inactive showed significantly lower EQ-5D relative to the manager/professional reference, while remaining occupational contrasts were not significant. Unmet medical need remained the single largest Part-2 predictor (β = −0.0269, 95% CI [−0.038, −0.016], *p* < 0.001), with a magnitude approaching the lower-bound MID threshold and reinforcing its dual role across both parts of the model.

**Figure 6:**
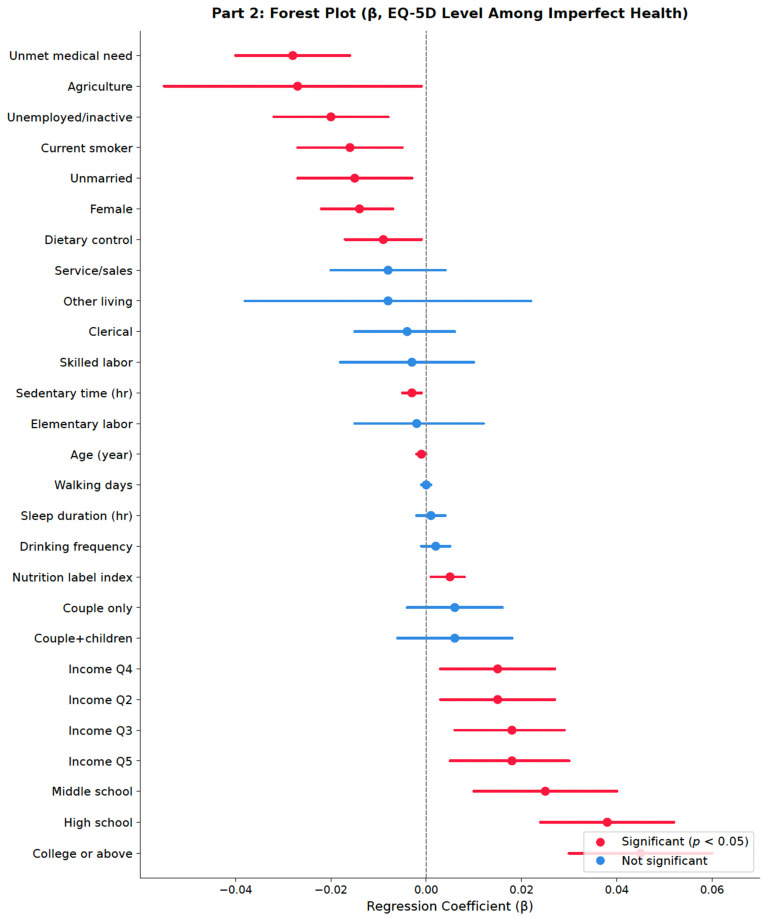
Forest Plot(2). Note: Forest plot of regression coefficients from the Part 2 survey-weighted OLS regression predicting EQ-5D utility among respondents with EQ-5D < 1.0 (n = 2713). Red markers indicate *p* < 0.05; blue markers indicate non-significance. The vertical dashed line at β = 0 represents the null. Reference categories: elementary or below (education); Q1 (income); manager/professional (occupation); living alone (living arrangement).

Figure 6 presents the Part 2 results in forest plot form. Unmet medical need (β = −0.027) was the largest negative predictor of EQ-5D utility among respondents with imperfect health, followed by unemployment, current smoking, unmarried status, female sex, and active dietary control. The nutrition label use index (β = 0.0047), education contrasts above the reference, and income quintiles Q2–Q5 were positive and statistically significant. Drinking frequency was the only behavioral covariate with a significant positive Part 1 association (Figure 5), although the coefficient did not persist in Part 2 (Figure 6), a pattern consistent with reverse selection—individuals with chronic conditions or dietary restrictions reduce alcohol intake.

### 4.3. Robustness Checks

To assess the robustness of the two-part model findings, an alternative OLS regression model was estimated on the full analytical sample (N = 5215) incorporating the same independent variables and complex survey design adjustments. This full-sample OLS model yielded an R-squared value of 0.166 and a positive, statistically significant coefficient for nutrition label use index (β = 0.0041, 95% CI [0.0014, 0.0068], *p* = 0.003), confirming that the positive association between nutrition label use and HRQoL is not an artifact of the two-part model specification or the restriction to the imperfect-health subsample. The direction and significance of all other key predictors—female gender, age, education, income, occupational class, smoking, sedentary time, and unmet medical need—were consistent between the full-sample OLS and the Part 2-restricted OLS, providing additional confidence in the stability of findings.

Multicollinearity diagnostics confirmed the absence of problematic collinearity: all VIF values were below 5.0, with the highest GVIF^(1/2Df) value of 1.737 observed for age, reflecting its expected moderate correlations with education and income but well within acceptable bounds. The collinearity between nutrition label use index and education (r = 0.412) and age (r = −0.410) is noteworthy, but the VIF diagnostics confirm that these correlations do not compromise the precision of the nutrition label coefficient to a degree that would render the estimate unreliable.

### 4.4. Sensitivity Analyses

#### 4.4.1. Decomposition of the Composite Index

Because the composite index aggregates three conceptually related but distinct components—awareness, use, and purchase influence—under an equal-weighting assumption, two alternative specifications were examined. In a joint specification all three binary indicators were entered simultaneously in place of the index. In a single-component specification, each indicator was entered alone, replacing the composite, with the full control-variable set retained. Table 7 summarizes the estimated focal coefficients across these specifications and the additional sensitivity analyses described below.

**Figure 7:**
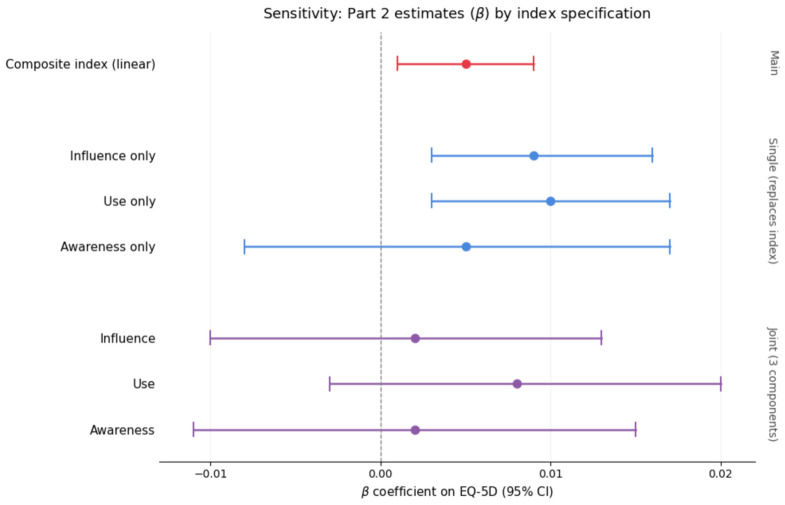
Part-2 EQ-5D estimates. Note. The signal in the composite specification originates predominantly in the use and influence components.

When the three binary components were entered jointly, none reached statistical significance in either Part 1 or Part 2 (joint Part-2 estimates: awareness β = 0.00215, *p* = 0.737; use β = 0.00836, *p* = 0.153; influence β = 0.00185, *p* = 0.745), reflecting collinearity among the cumulative engagement stages. When entered separately, however, both use (β = 0.0102, *p* = 0.005) and purchase influence (β = 0.0095, *p* = 0.008) showed statistically significant positive associations with EQ-5D in the imperfect-health subsample, while awareness alone did not (β = 0.00494, *p* = 0.432). The pattern is consistent with the cumulative-stage interpretation of the index: the empirical signal carried by the composite is concentrated in the use and purchase-influence stages rather than in mere awareness, and the composite specification preserves statistical power that is otherwise lost to collinearity when the three components are estimated simultaneously. Figure 7 displays the corresponding Part-2 forest plot.

Part-2 EQ-5D estimates (β with 95% CI) under three specifications of the nutrition-label-use index: composite (continuous), single-component (each indicator replacing the composite), and joint (all three components entered together).

#### 4.4.2. Ordinal Treatment of the Index

**Figure 8:**
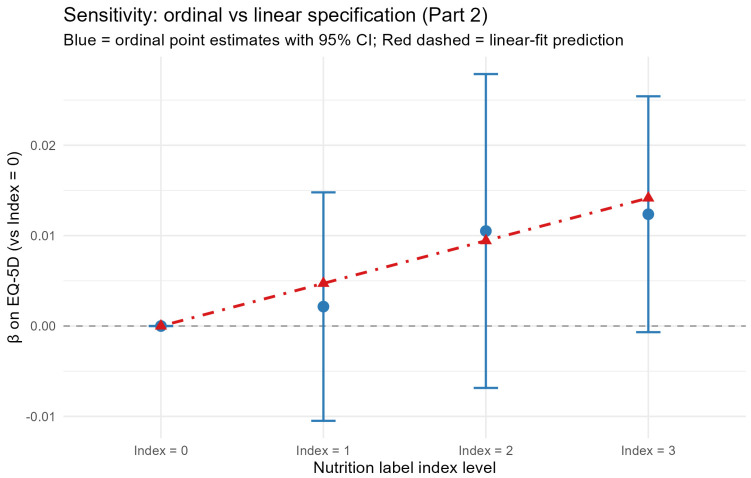
Ordinal versus linear specification of the nutrition-label-use index (Part 2 OLS). Note. Blue circles with 95% CI bars are the ordinal point estimates relative to Index = 0; the red dot-dashed line traces the linear-fit prediction (β × index level) using the main-analysis coefficient.

When the index was entered as a four-level factor with Index = 0 as the reference, the Part-2 EQ-5D point estimates increased monotonically across the three non-reference levels (Index 1: β = 0.0022, *p* = 0.737; Index 2: β = 0.0105, *p* = 0.233; Index 3: β = 0.0124, *p* = 0.063). Although individual category contrasts did not all reach significance—partly reflecting reduced cell sizes—an orthogonal-polynomial decomposition of the ordered factor produced a statistically significant linear component (β = 0.0102, *p* = 0.040) with non-significant quadratic (*p* = 0.974) and cubic (*p* = 0.458) components. This pattern supports the linear specification of the index used in the main analysis. Figure 8 overlays the ordinal point estimates against the linear-fit prediction and shows close correspondence.

#### 4.4.3. Multiple Imputation of Missing Data

**Figure 9:**
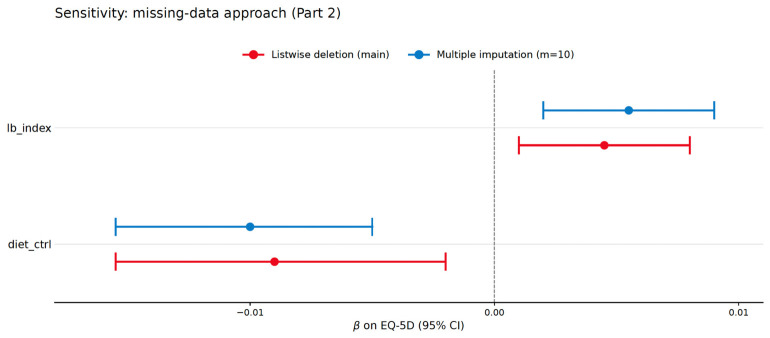
Missing-data sensitivity: focal Part-2 OLS coefficients under listwise deletion versus pooled multiple imputation (m = 10). Note. Red = listwise-deletion main analysis (n = 2713). Blue = pooled estimates from multiple imputation with m = 10 (n = 5859 pre-imputation; complex-survey design retained within each imputed dataset). The multiple imputation procedure was applied to the 5859 age-eligible respondents with complete data on the EQ-5D outcome (5982 age-eligible respondents minus 123 with item non-response on one or more EQ-5D dimensions); covariates with item non-response were imputed within this restricted sample under MAR assumptions.

To assess the robustness of the main findings under plausible missing-at-random departures from the listwise-deletion assumption, Part 1 and Part 2 were re-estimated after multiply imputing the analytical variables. Ten imputations were generated using the MICE algorithm (predictive mean matching for continuous variables and logistic/polytomous logistic regression for categorical variables); each imputed dataset was fit using the same complex-survey design as the main analysis, and the resulting coefficients were combined using Rubin’s rules. The pooled focal coefficients reproduced the listwise results closely: Part 1 lb_index OR = 1.067, 95% CI [0.997, 1.142]; Part 2 lb_index β = 0.00556, 95% CI [0.00220, 0.00892]; Part 2 diet_ctrl β = −0.0106, 95% CI [−0.0175, −0.00372]. The Part-2 label-use coefficient is, if anything, slightly larger under multiple imputation than under listwise deletion, indicating that the listwise-deletion estimate is not inflated by the over-representation of older or less-educated respondents among excluded cases. Figure 9 visualizes the comparison.

#### 4.4.4. Part-2 OLS Diagnostics and Robust Standard Errors

Formal residual diagnostics on the Part-2 model (n = 2713 with EQ-5D < 1.0) indicated departures from the OLS regularity conditions. A Shapiro–Wilk test on the residuals rejected normality (W = 0.85, *p* < 0.001), and a Breusch–Pagan test on the corresponding unweighted OLS specification rejected homoscedasticity (BP = 137.0, df = 27, *p* < 0.001). Full residual diagnostic plots and the complete HC3 comparison are provided in Supplementary Figures S1 and S2 and Supplementary Tables S1 and S2. These departures are unsurprising given that EQ-5D, even after excluding the ceiling, remains a bounded outcome with right-skew. To assess inferential robustness under these violations, the Part-2 OLS specification was re-estimated on the unweighted subsample with HC3 heteroscedasticity-consistent standard errors. The focal nutrition-label-use coefficient remained statistically significant (β = 0.00379, HC3 SE = 0.00185, *p* = 0.040), although attenuated relative to the survey-weighted main estimate of β = 0.00472 (*p* = 0.006). The dietary-control coefficient was likewise robust (β = −0.0104, *p* = 0.006). The convergent inferential conclusion across (a) survey-weighted OLS, (b) unweighted OLS with HC3 robust SEs, (c) the full-sample OLS specification reported in Section 4.3, and (d) the multiply-imputed estimation in Section 4.4.3 supports the stability of the main finding.

#### 4.4.5. Heterogeneity by Dietary Control

Whether dietary control modifies the nutrition-label-use → EQ-5D association was tested by adding a lb_index × diet_ctrl interaction term to the Part-2 specification. The interaction was non-significant (β = 0.00098, *p* = 0.735), implying that the magnitude of the label-use association does not differ meaningfully between respondents who do and do not currently report dietary control. Stratified estimates were directionally consistent: among respondents not currently controlling their diet (the larger stratum), lb_index β = 0.00512 (95% CI [0.00146, 0.00877], *p* = 0.006); among respondents currently controlling their diet, lb_index β = 0.00447 (95% CI [−0.00171, 0.01065], *p* = 0.155), with the wider confidence interval reflecting reduced sub-sample size. Together, these results suggest a relatively homogeneous label-use association across dietary-control status rather than an effect concentrated in disease-management subpopulations.

## 5. Discussion

### 5.1. Theoretical Implications

This study offers a cautious observational contribution to the literature on dietary behavior, health-related quality of life, and the social determinants of health in the Korean context. The central finding—that nutrition label use is modestly and positively associated with HRQoL among Korean adults who do not report perfect health—extends the descriptive evidence on diet-literacy-related correlates of generic health utility. Prior research has primarily examined the relationship between nutrition label use and dietary choices or proximal nutritional outcomes, such as energy intake and sodium consumption, but has rarely extended this line of inquiry to generic preference-based HRQoL measures [8,9,13]. The present findings suggest that nutrition label engagement may be a relevant behavioral marker associated with multidimensional health utility, rather than evidence that nutrition label use itself improves HRQoL. Notably, this association was observed under Korea’s current back-of-pack labeling system, where consumers must actively locate and interpret numerical nutritional information without the benefit of front-of-pack interpretive aids—a regulatory context that may attenuate the observed effect relative to what might be expected under more accessible labeling formats [19].

Because the analysis rests on a single cross-sectional wave of KNHANES, all interpretations should be read as associative and hypothesis-generating rather than as evidence of causal effects. The empirical claim supported by the present analysis is restricted to a small but statistically robust positive association between cumulative nutrition-label engagement and EQ-5D utility scores among Korean adults with imperfect health, conditional on the measured covariates.

#### 5.1.1. Practical Significance of the Nutrition Label Effect

While the positive association between nutrition label use and EQ-5D was statistically significant, its practical magnitude should be interpreted cautiously. In Part 2, each one-unit increase in the nutrition label use index was associated with a 0.0047-unit increase in EQ-5D utility. Across the full range of the index from 0 to 3, this corresponds to a maximum difference of 0.0141 units. This magnitude falls below commonly cited minimally important difference thresholds for the EQ-5D-3L: Pickard and colleagues [36] estimated MID values ranging from 0.03 to 0.05, while Walters and Brazier [37] eported a mean MID of approximately 0.074 across multiple patient groups. Thus, the observed association is unlikely to represent a clinically meaningful difference at the individual level.

At the population level, the finding remains relevant as a statistically detectable association in nationally representative data, but it should not be interpreted as evidence that increasing nutrition label use would directly improve HRQoL. The coefficient may reflect nutrition label engagement itself, but it may also capture broader health-oriented characteristics, such as health consciousness, self-regulatory orientation, health literacy, or general health-seeking behavior. Therefore, the practical significance of the association is best understood as modest and exploratory. Longitudinal or quasi-experimental research would be required to determine whether changes in nutrition label engagement precede meaningful changes in dietary quality or HRQoL.

#### 5.1.2. Differential Patterns Across the Two-Part Model

The differential pattern of nutrition label associations across the two parts of the model is theoretically informative. In Part 1, nutrition label use was not significantly associated with achieving perfect health, while in Part 2, it was significantly associated with EQ-5D level among those with imperfect health. This pattern suggests that nutrition label use is associated with modest differences in health utility within the imperfect-health range, but it does not differentiate the probability of reporting perfect health in Part 1.

This interpretation is consistent with a threshold model of health: the transition to perfect health on the EQ-5D requires the absence of any problems across all five dimensions, a stringent criterion that depends on factors—particularly biological aging, genetic predisposition, and accumulated chronic disease burden—that are less amenable to modification by dietary behavior. Within the more heterogeneous imperfect-health range, however, behavioral indicators such as nutrition label use may be correlated with modest variation in health utility. This finding underscores the methodological importance of the two-part model approach: a conventional OLS model applied to the full sample would have yielded a less precise estimate of the nutrition label effect by conflating these two qualitatively distinct processes.

#### 5.1.3. The Dietary Control Paradox

The dietary control paradox—whereby dietary self-management is negatively associated with HRQoL in both parts of the model—provides important lessons for the interpretation of cross-sectional behavioral data. From a causal perspective, this finding is most plausibly explained by reverse causality: individuals with health conditions characterized by lower HRQoL (e.g., diabetes, hypertension, obesity) are prescribed or self-motivated to modify their diets, generating a positive correlation between disease burden and dietary control. The cross-sectional design of KNHANES cannot establish the temporal sequence of dietary control and HRQoL change, making it impossible to distinguish this reverse causality from a genuine adverse effect of dietary restriction on health utility—perhaps through the psychological burden of restrictive diets, social disruption of shared eating practices, or dietary inadequacy resulting from overly restrictive self-management. Future longitudinal research is needed to disentangle these competing explanations.

#### 5.1.4. Social Gradient in HRQoL

The monotonic education and income gradients evident in Part 2 align with the social-determinants-of-health framework articulated by Marmot and by Braveman and colleagues [26,27]: HRQoL advantages emerge at every step up the social hierarchy rather than only above a poverty threshold, and the strong correlation of education with both nutrition label use (r = 0.412) and EQ-5D (r = 0.300) is consistent with education operating as an upstream determinant generating correlated advantages in health literacy, dietary engagement, and health utility—an interpretation that reinforces prior Korean evidence [21,28] and underscores the limits of behavioral-level interventions undertaken in isolation from structural reform. Within this gradient, unmet medical need stands out as the single largest predictor in both parts of the model: even within Korea’s near-universally insured population, geographic, financial, and literacy barriers can prevent timely care utilization [33], and the 7.9% prevalence of unmet need observed here carries an EQ-5D decrement (β = −0.0269) that approaches the lower-bound MID threshold for a single self-reported access-barrier indicator.

### 5.2. Practical Implications

Two areas of policy relevance follow from the present cross-sectional associations, with appropriate caveats about the limits of causal inference from observational data. First, the positive label–HRQoL association observed under Korea’s existing back-of-pack regime—where label engagement requires active consumer effort to locate and interpret numerical nutritional information—is consistent with, though not direct evidence for, the hypothesis that more accessible front-of-pack interpretive formats such as traffic-light or warning-label systems are shown to be effective in international evidence [14,19] may be associated with stronger expression of the diet-literacy–HRQoL pattern documented here. The pronounced age gradient in label use (r = −0.410) further suggests that older consumers face systematic visual, cognitive, and literacy barriers under existing label formats; age-targeted health-literacy interventions and simplified-format labels with larger fonts represent complementary strategies [11,15]. Second, the magnitude of the unmet-medical-need coefficient—the largest single Part-2 predictor and one approaching the lower-bound MID threshold—highlights structural healthcare access as the most consequential modifiable correlate of HRQoL identified in either part of the model, with out-of-pocket cost burden, geographic distribution of services, and informational and navigational barriers each plausibly contributing to the observed disparity [33].

Two population subgroups warrant integrated attention. Agricultural workers and the unemployed/economically inactive both exhibit significantly lower EQ-5D after comprehensive covariate adjustment, reflecting compounded exposure to physically demanding or hazardous working conditions, geographic remoteness from healthcare facilities, limited occupational health protections, agricultural-chemical exposure, and—for the unemployed/inactive subgroup—the psychological and material stressors of economic insecurity alongside the loss of social and identity benefits associated with paid employment. The persistent gender gap that survives full covariate adjustment likewise points to women’s disproportionate burden of pain and psychological distress, visible in the EQ-5D pain/discomfort and anxiety/depression dimensions; the dual burden of paid employment and domestic care responsibilities in Korea, combined with persistent gender pay gaps and occupational segregation, creates conditions that individual behavioral interventions are unlikely to address in isolation, implicating instead structural reforms in labor policy, social-support systems, and caregiving infrastructure.

International comparison is constrained by methodological heterogeneity across labeling regimes, EQ-5D instrument versions, value-set derivations, and survey-weighting conventions, but several reference points are informative. The Cheng et al. [20] meta-analysis estimated East and Southeast Asian EQ-5D-3L ceiling shares around 56%, somewhat higher than European pooled estimates near 40%; the present sample’s 48% ceiling share aligns with the East Asian regional pattern and reinforces the methodological case for two-part modeling in this regional context, where direct cross-regional comparisons of utility means or coefficients should treat ceiling-effect handling as a substantive analytical decision rather than as a routine technical detail. Direction-of-association evidence on label use and dietary behavior in non-Korean populations [13,14,15,19] is broadly consistent with the positive cross-sectional label–outcome association documented here, although a quantitative cross-regime comparison of label–HRQoL coefficients would require harmonized exposure measurement that the present single-country observational design cannot provide—a useful direction for future cross-national collaborative research.

### 5.3. Limitations and Future Directions

Several limitations of this study merit acknowledgment. First, the cross-sectional design of KNHANES 2024 precludes causal inference. Observed associations between nutrition label use and HRQoL may reflect unmeasured confounders or reverse causality: individuals with higher HRQoL may have greater cognitive and motivational resources for engaging with nutrition labels, generating a reverse-causal pattern that cross-sectional analysis cannot definitively rule out. While the reverse causality concern is explicitly modeled for dietary control (which shows the expected negative association consistent with health-impairment-driven dietary modification), the same concern applies, albeit with different theoretical expectations, to nutrition label use. Future longitudinal research using repeated KNHANES waves is needed to establish temporal ordering.

Second, all variables—including the dependent variable, main independent variables, and health behavior measures—rely on self-report, which may be subject to social desirability bias, recall error, and measurement imprecision. The nutrition label use index measures self-reported engagement rather than objectively verified behavior, and may overestimate actual label consultation among health-conscious respondents. The formative structure of the index—capturing three conceptually distinct stages of label engagement—partially mitigates concerns about measurement validity, but objective verification through eye-tracking or purchase receipt analysis would strengthen future research.

Third, the analytical specification used here does not include diagnosed chronic-disease status (e.g., hypertension, diabetes, dyslipidemia, ischemic heart disease, stroke) or screening-level mental-health symptomatology, both of which are plausibly associated with nutrition-label engagement and with HRQoL. Diagnosed chronic disease may shape label engagement through clinician advice, symptom-driven information seeking, and disease-specific dietary guidance, and may shape HRQoL through accumulated disease burden; the strongly negative dietary-control coefficient observed in both parts of the model is consistent with this pathway. The dietary-control item retained in the specification—affirmatively coded for respondents currently modifying their diet for medical, weight-management, or general-health reasons—partially absorbs the chronic-disease channel even in the absence of a direct diagnostic adjustment, in that respondents under disease-management dietary modification are over-represented within the affirmatively coded group. Mental-health symptomatology loads directly on the EQ-5D anxiety/depression dimension and may co-vary with health-conscious behavior including label engagement. The present analysis was designed to focus on the dietary-behavior and social-determinant pathways central to the research question; substantive incorporation of chronic-disease and mental-health adjustment, which would entail its own theoretical framing and analytical structure, is reserved for a planned follow-up study explicitly designed to test these channels. Residual confounding from these sources, as well as from health and nutrition literacy and food-environment characteristics, cannot be ruled out.

Fourth, of the 6997 source respondents, 1015 were excluded on the 19–80 age-eligibility criterion and a further 767 were excluded by listwise deletion of cases with item non-response on the analytical variables, leaving the present analytical sample of N = 5215. The listwise-deletion step (767 of 5982 age-eligible respondents, or 12.8%) raised the possibility of selection bias, with excluded cases skewing toward older and less-educated respondents. The multiple-imputation re-analysis reported in Section 4.4.3—m = 10 imputations using MICE with predictive mean matching for continuous variables and polytomous logistic regression for categorical variables, pooled under Rubin’s rules with the complex-survey design retained within each imputed dataset—produced a Part-2 nutrition-label-use coefficient (β = 0.00556) marginally larger than the listwise-deletion estimate (β = 0.00472), which supports the robustness of the focal finding under missing-at-random assumptions. The possibility of missing-not-at-random mechanisms cannot be definitively excluded without external validation data and remains a residual limitation.

Fifth, the dietary control item captures a heterogeneous category of behaviors—from physician-prescribed therapeutic diets for diabetes management to self-directed caloric restriction for weight management—without distinguishing the type, intensity, or duration of dietary modification. This heterogeneity may attenuate or distort the estimated association between dietary control and HRQoL, and future research should employ more granular dietary behavior measures.

Sixth, while the study includes a comprehensive set of sociodemographic and behavioral covariates, residual confounding from unmeasured variables cannot be excluded. In particular, the nutrition label use index may partly reflect broader health consciousness, self-regulatory orientation, health literacy, or general health-seeking tendencies, rather than the independent contribution of nutrition label use alone. Food environment characteristics, such as proximity to supermarkets versus convenience stores, may also shape both label use and HRQoL-related behaviors. Instrumental variable approaches, natural experiments, or longitudinal designs would provide stronger causal leverage. The reported associations should therefore be interpreted as exploratory estimates of the relationship between nutrition-related information engagement and HRQoL, rather than as confirmatory evidence of a diet-literacy-to-HRQoL pathway.

Future research should prioritize three directions. First, longitudinal panel designs using repeated KNHANES waves would establish temporal ordering between nutrition label use, dietary quality change, and HRQoL trajectories, enabling more credible assessment of causal relationships. Second, mediation analyses incorporating 24 h dietary recall data from KNHANES would permit direct testing of whether the label–HRQoL association operates through improved dietary quality—the central but untested mediator in the conceptual framework. Third, cross-national comparative studies across countries with different labeling systems, such as Korea’s back-of-pack system, Chile’s mandatory FOP warnings, and France’s Nutri-Score, would help clarify whether labeling format is associated with differences in nutrition-label engagement, dietary behavior, and health utility.

## 6. Conclusions

This study makes three principal contributions to the literature on dietary behavior and population health. First, it provides nationally representative cross-sectional evidence that nutrition label utilization is modestly associated with HRQoL, as measured by a validated preference-based utility instrument, the EQ-5D-3L, in an East Asian population sample. The finding that nutrition label engagement is associated with higher EQ-5D scores among adults with imperfect health (β = 0.0047, *p* = 0.006) extends the descriptive evidence base on diet-literacy-related correlates of health utility from proximal dietary endpoints to a multidimensional generic-utility instrument. However, the effect size was small and below commonly cited clinical MID thresholds. Therefore, this finding should be interpreted as an exploratory association rather than as evidence that nutrition label use independently improves HRQoL.

Second, the study demonstrates the methodological value of the two-part model for EQ-5D analyses in community-dwelling populations where ceiling effects are prevalent. The differential patterns across Parts 1 and 2—with nutrition label use significant only in Part 2—suggest that the correlates of reporting perfect health may differ from the correlates of health utility among those with imperfect health. These patterns could have been obscured by conventional OLS regression.

Third, the comprehensive covariate structure highlights the strong associations of social structural determinants—particularly education, income, and unmet medical need—with HRQoL, situating behavioral indicators such as nutrition label use within their broader population health context. The finding that unmet medical need was the strongest correlateacross both model parts reinforces the importance of healthcare access as a structural determinant of population health utility, while also indicating that behavioral indicators should be interpreted alongside social and structural conditions.

These cross-sectional findings may inform future research and cautious policy discussion in Korea and comparable East Asian contexts undergoing nutritional transition, but they do not provide direct evidence for specific regulatory effects. Given that Korea has not yet adopted a mandatory front-of-pack interpretive nutrition labeling system, future studies could examine whether more accessible labeling formats, such as traffic-light systems or warning labels, are associated with stronger nutrition-label engagement and improved dietary choices. The strong age gradient in label use also suggests the need for further research on whether age-targeted health literacy interventions can improve label comprehension among older populations. Future longitudinal research using repeated KNHANES waves, mediation analyses incorporating dietary quality data, and cross-national comparative studies across labeling systems are needed to establish temporal ordering, test potential mechanisms, and identify the conditions under which nutrition labeling is associated with meaningful differences in dietary behavior and HRQoL.

## Figures and Tables

**Table 1:** Conceptual and operational definitions of analytical variables.

Construct	Conceptual Definition	Operational Measurement	Justification
Health-related quality of life	Generic preference-based health utility integrating mobility, self-care, usual activities, pain/discomfort, and anxiety/depression dimensions	EQ-5D-3L scored with Korean time-trade-off weights [5]; empirical range −0.013 to 1.000	Validated for Korean adults [5,6] and standard outcome in Korean and international population health and cost-utility research [4]
Nutrition label use	Cumulative engagement with packaged-food nutrition information across awareness, behavioral consultation, and purchase influence	Composite index (0–3) summing three binary KNHANES items (LK_LB_CO, LK_LB_US, LK_LB_EF) administered under a conditional skip pattern	Formative-indicator framework appropriate for an inherently cumulative-stage construct [10,34]; consistent with prior population-survey operationalization of label engagement [8,10]
Dietary self-management	Intentional, health-motivated dietary modification undertaken for medical, weight-management, or general-health reasons	Single binary KNHANES item N_DIET (“currently controlling diet”; yes = 1)	Captures self-reported behavioral engagement at a level consistent with health-behavior surveillance practice; heterogeneity in motivation and intensity acknowledged as a measurement limitation in Section 5.3
Education	Highest formal educational attainment as a marker of accumulated cognitive, informational, and health-literacy resources	Four ordered categories (≤elementary/middle/high/≥college); ≤elementary as reference	Standard Korean SES marker; correlates with health literacy and HRQoL in prior KNHANES and KCHS analyses [21,28]
Income	Household material resources adjusted for household composition	Equivalized household income quintile (Q1 lowest, reference; Q5 highest)	Quintile categorization is the conventional operationalization in KNHANES analyses, capturing non-linear gradient effects observed in prior Korean evidence [22]
Occupational class	Occupational position as a marker of working-condition exposure, social position, and identity-based resources	Seven-category Korean Standard Classification of Occupations (KSCO) coding; manager/professional as reference	KSCO is the official Korean occupational-coding scheme; widely used in Korean SES and health-equity research [12]
Marital status	Partnership-derived social and economic support	Married versus unmarried (never married, divorced, widowed combined)	Pragmatic dichotomization given the Korean policy and survey convention; nuanced unmarried subcategories collapse out of statistical-power necessity in subgroup analyses
Living arrangement	Household co-residence configuration relevant to caregiving and social support	Living alone (reference), couple only, couple with children, other	Household-level living arrangement is independently associated with HRQoL net of marital status
Smoking	Tobacco-related modifiable behavioral risk	Current smoker versus non-smoker (binary)	Robustly associated with multiple HRQoL dimensions [30]
Drinking frequency	Alcohol-consumption modifiable behavioral risk	Seven-point ordinal scale (0 = non-drinker; 6 = daily)	Standard KNHANES operationalization; the alcohol–HRQoL relationship is contested [7] and the variable is retained for adjustment purposes
Sedentary time	Daily sitting time independent of physical activity	Continuous hours per day	Independently associated with cardiometabolic and HRQoL outcomes [31]
Walking days	Light-to-moderate physical activity exposure	Days per week of any walking ≥ 10 min (0–7)	KNHANES standard operationalization compatible with international physical-activity surveillance
Sleep duration	Habitual nightly sleep exposure	Average daily sleep hours, computed from KNHANES bedtime and wake-time items as (weekday sleep × 5 + weekend sleep × 2)/7	Both short and long sleep have been associated with adverse HRQoL [32]
Unmet medical need	Self-reported failure to obtain medically needed care	Single binary KNHANES item over a 12-month reference window (yes = 1)	Operationalizes the Penchansky–Thomas access framework [33]; widely used in Korean health-equity research
Chronic-disease status (sensitivity only; see §5.3)	Aggregate burden of major non-communicable conditions associated with HRQoL impairment	Binary composite of physician-diagnosis indicators (HE_HP, HE_DM, HE_HCHOL, DI1_dg, DI3_dg)	Captures the principal NCD profile relevant to dietary-behavior research; reserved for sensitivity reanalysis discussed in Section 5.3
Depression screening (sensitivity only; see §5.3)	Severity of depressive symptomatology over the prior two-week reference period	PHQ-9 sum score (0–27)	Loads directly on the EQ-5D anxiety/depression dimension; reserved for sensitivity reanalysis discussed in Section 5.3

**Table 2:** Weighted sample characteristics of the analytical sample (N = 5215).

Variable	Weighted Statistic
Age, mean (SD)	49.66 (16.94)
Female (%)	50.3
Education: elementary or below (%)	11.2
Education: middle school (%)	8.3
Education: high school (%)	33.5
Education: college or above (%)	47.1
Income Q1/Q2/Q3/Q4/Q5 (%)	18.5/19.8/20.4/20.3/21.0
Occupation: manager/professional (%)	18.6
Occupation: clerical (%)	12.3
Occupation: service/sales (%)	15.1
Occupation: agriculture (%)	1.9
Occupation: skilled labor (%)	10.8
Occupation: elementary labor (%)	8.6
Occupation: unemployed/inactive (%)	32.8
Married (%)	74.5
Living alone/couple only/couple + children/other (%)	22.1/60.5/16.8/0.6
Current smoker (%)	15.3
Dietary control, yes (%)	32.6
Unmet medical need, yes (%)	7.9
EQ-5D utility index, mean (SD)	0.928 (0.096)
EQ-5D = 1.0, ceiling (%)	48.0
Nutrition label use index (0–3), mean (SD)	1.591 (1.081)
Drinking frequency (0–6), mean (SD)	2.943 (1.697)
Daily sedentary time (hours), mean (SD)	9.197 (3.141)
Weekly walking days (0–7), mean (SD)	2.684 (2.458)
Average daily sleep (hours), mean (SD)	7.418 (1.210)

Note: Statistics are weighted to represent the Korean adult civilian population aged 19–80. EQ-5D utility scores reflect Korean TTO weights [5]. Nutrition label use index = sum of three binary items (awareness, use, purchase influence). Income quintiles based on equivalized household income.

**Table 3:** Distributional range (min, max) of key continuous variables (weighted, N = 5215).

Variable	Mean	SD	Min	Max
EQ-5D utility index	0.928	0.096	−0.013	1.000
Nutrition label use index	1.591	1.081	0	3
Age (years)	49.66	16.94	19	80
Drinking frequency (0–6)	2.943	1.697	0	6
Daily sedentary time (hours)	9.197	3.141	0	24
Weekly walking days (0–7)	2.684	2.458	0	7
Average daily sleep (hours)	7.418	1.210	3.0	12.0

Note: Drinking frequency: 0 = non-drinker; 1 = 1–11 times/year; 2 = 1–3 times/month; 3 = once/week; 4 = 2–3 times/week; 5 = 4–5 times/week; 6 = daily.

**Table 4:** Pearson correlations among key study variables.

Variable	1	2	3	4	5	6	7	8
1. EQ-5D	—							
2. Nutrition label index	0.166 **	—						
3. Age	−0.270 **	−0.410 **	—					
4. Education	0.300 **	0.412 **	−0.479 **	—				
5. Income quintile	0.088 **	0.109 **	−0.099 **	0.217 **	—			
6. Unmet medical need	−0.161 **	−0.020	0.036 **	−0.071 **	−0.074 **	—		
7. Sedentary time	−0.079 **	−0.030 *	0.098 **	−0.109 **	−0.036 **	0.033 *	—	
8. Drinking frequency	0.175 **	0.026	−0.152 **	0.052 **	0.096 **	−0.044 **	−0.009	—

Note. * *p* < 0.05. ** *p* < 0.01. Education and income quintile treated as ordinal for correlation.

**Table 5:** Part 1: Survey-weighted logistic regression predicting perfect health (EQ-5D = 1.0 vs. <1.0; N = 5215).

Variable	OR	95% CI	*p*
Nutrition label use index	1.057	[0.985, 1.134]	0.124
Dietary control (yes)	0.819	[0.705, 0.951]	0.009
Female	0.584	[0.498, 0.684]	<0.001
Age (years)	0.985	[0.977, 0.992]	<0.001
Education: middle school	1.058	[0.820, 1.364]	0.662
Education: high school	1.204	[0.954, 1.520]	0.117
Education: college or above	1.352	[1.053, 1.736]	0.019
Income Q2	1.095	[0.884, 1.356]	0.403
Income Q3	1.013	[0.817, 1.257]	0.896
Income Q4	1.112	[0.910, 1.359]	0.299
Income Q5	1.164	[0.911, 1.488]	0.229
Occupation: clerical	1.118	[0.882, 1.416]	0.356
Occupation: service/sales	0.827	[0.664, 1.029]	0.095
Occupation: agriculture	0.868	[0.611, 1.233]	0.451
Occupation: skilled labor	0.967	[0.745, 1.255]	0.818
Occupation: elementary labor	0.895	[0.677, 1.182]	0.439
Occupation: unemployed/inactive	0.833	[0.691, 1.005]	0.058
Unmarried	0.890	[0.690, 1.148]	0.386
Living: couple only	1.058	[0.907, 1.234]	0.470
Living: couple with children	0.896	[0.720, 1.115]	0.337
Living: other	0.934	[0.457, 1.909]	0.864
Current smoker	0.778	[0.648, 0.935]	0.008
Drinking frequency	1.047	[1.004, 1.092]	0.030
Sedentary time (hours)	0.973	[0.955, 0.992]	0.005
Walking days (0–7)	1.018	[0.991, 1.046]	0.191
Sleep duration (hours)	1.050	[0.991, 1.113]	0.098
Unmet medical need (yes)	0.315	[0.248, 0.401]	<0.001

Note. *p* < 0.05. OR = odds ratio; CI = confidence interval. Survey-weighted logistic regression with complex survey design (kstrata, psu, wt_tot). Quasibinomial family.

**Table 6:** Part 2: Survey-weighted OLS regression predicting EQ-5D score among those with imperfect health (EQ-5D < 1.0; n = 2713).

Variable	β	95% CI	*p*
Nutrition label use index	0.0047	[0.0013, 0.0081]	0.006
Dietary control (yes)	−0.0091	[−0.0165, −0.0017]	0.016
Female	−0.0140	[−0.0208, −0.0072]	<0.001
Age (years)	−0.0009	[−0.0012, −0.0006]	<0.001
Education: middle school	0.0251	[0.0103, 0.0398]	0.001
Education: high school	0.0379	[0.0235, 0.0522]	<0.001
Education: college or above	0.0456	[0.0307, 0.0605]	<0.001
Income Q2	0.0149	[0.0044, 0.0254]	0.006
Income Q3	0.0173	[0.0065, 0.0281]	0.002
Income Q4	0.0148	[0.0041, 0.0255]	0.007
Income Q5	0.0174	[0.0048, 0.0300]	0.007
Occupation: clerical	−0.0039	[−0.0141, 0.0063]	0.477
Occupation: service/sales	−0.0083	[−0.0190, 0.0024]	0.126
Occupation: agriculture	−0.0252	[−0.0498, −0.0005]	0.045
Occupation: skilled labor	−0.0033	[−0.0161, 0.0095]	0.643
Occupation: elementary labor	−0.0009	[−0.0143, 0.0125]	0.898
Occupation: unemployed/inactive	−0.0194	[−0.0295, −0.0092]	<0.001
Unmarried	−0.0145	[−0.0254, −0.0035]	0.010
Living: couple only	0.0061	[−0.0037, 0.0159]	0.223
Living: couple with children	0.0062	[−0.0045, 0.0169]	0.251
Living: other	−0.0077	[−0.0372, 0.0218]	0.605
Current smoker	−0.0152	[−0.0258, −0.0047]	0.005
Drinking frequency	0.0014	[−0.0004, 0.0032]	0.133
Sedentary time (hours)	−0.0029	[−0.0042, −0.0015]	<0.001
Walking days (0–7)	0.0001	[−0.0007, 0.0009]	0.865
Sleep duration (hours)	0.0010	[−0.0019, 0.0039]	0.490
Unmet medical need (yes)	−0.0269	[−0.0383, −0.0155]	<0.001

Note. *p* < 0.05. β = unstandardized regression coefficient; CI = confidence interval. R^2^ = 0.180. Survey-weighted OLS with complex design (kstrata, psu, wt_tot).

**Table 7:** Sensitivity analyses for the nutrition-label-use specification.

Specification	Coefficient	β/OR	95% CI	*p*
Main: index continuous (Part 2 OLS)	lb_index	β = 0.00472	[0.00135, 0.00809]	0.006
Joint 3 components (Part 2)	Awareness	β = 0.00215	[−0.0105, 0.0148]	0.737
	Use	β = 0.00836	[−0.00315, 0.0199]	0.153
	Influence	β = 0.00185	[−0.00937, 0.0131]	0.745
Single component (Part 2; replaces index)	Awareness only	β = 0.00494	[−0.00747, 0.0174]	0.432
	Use only	β = 0.0102	[0.00308, 0.0173]	0.005
	Influence only	β = 0.0095	[0.00252, 0.0165]	0.008
Ordinal index (Part 2; vs. Index 0)	Index 1	β = 0.00215	[−0.0105, 0.0148]	0.737
	Index 2	β = 0.0105	[−0.00685, 0.0279]	0.233
	Index 3	β = 0.0124	[−0.00068, 0.0254]	0.063
Polynomial trend test (Part 2; ordered)	Linear (.L)	β = 0.0102	[0.00046, 0.0199]	0.040
	Quadratic (.Q)	β = −0.00015	[−0.00939, 0.00909]	0.974
	Cubic (.C)	β = −0.00285	[−0.0104, 0.00472]	0.458
Multiple imputation (m = 10)	lb_index (Part 1)	OR = 1.067	[0.997, 1.142]	—
	lb_index (Part 2)	β = 0.00556	[0.00220, 0.00892]	0.001
HC3 robust SE (Part 2 OLS, unweighted)	lb_index	β = 0.00379	[—]	0.040
Diet-control × index interaction (Part 2)	lb_index × diet_ctrl	β = 0.00098	[−0.00472, 0.00668]	0.735
Diet-control stratified (Part 2)	diet_ctrl = 0	β = 0.00512	[0.00146, 0.00877]	0.006
	diet_ctrl = 1	β = 0.00447	[−0.00171, 0.01065]	0.155

Note. Part-1 estimates are odds ratios from survey-weighted logistic regression predicting EQ-5D = 1.0 (N = 5215). Part-2 estimates are unstandardized β coefficients from survey-weighted OLS restricted to EQ-5D < 1.0 (n = 2713) unless otherwise noted. The HC3 robust-SE row is from an unweighted OLS specification, which permits formal heteroscedasticity-consistent inference; the β attenuates relative to the survey-weighted main estimate but remains statistically significant at α = 0.05.

## Data Availability

The KNHANES 2024 data analyzed in this study are publicly available from the KNHANES website: https://knhanes.kdca.go.kr (accessed on 10 April 2026).

## Supplementary Materials

The following supporting information can be downloaded at: https://www.mdpi.com/article/10.3390/healthcare14101419/s1, Figure S1: Residuals-versus-fitted plot for the Part-2 OLS specification; Figure S2: Normal Q-Q plot of Part-2 residuals; Table S1: Part-2 diagnostic test results (Shapiro–Wilk, Breusch–Pagan); Table S2: HC3 robust standard errors versus survey-weighted Part-2 OLS coefficients (full coefficient vector); Table S3: STROBE Statement checklist for cross-sectional studies. All supplementary items are provided in the Supplementary Materials.
