# Supplementary material for: Nutrition Label Utilization, Dietary Self-Management, and Health-Related Quality of Life Among Korean Adults: A Two-Part Model Analysis of Nationally Representative Survey Data
Source: Healthcare (Basel). 2026 May 21;14(10):1419. doi: 10.3390/healthcare14101419 (PMC13205380; doi:10.3390/healthcare14101419)
Supplement: Supplementary file 1 [file healthcare-14-01419-s001.zip › healthcare-4283737-supplementary.pdf]

Supplementary Materials

Nutrition Label Utilization, Dietary Self-Management, and Health-Related Quality of Life Among Korean Adults: A Two-Part Model Analysis of Nationally Representative Survey Data

Yoonjin Lee — Konkuk University, Seoul, South Korea
leeyoonjin@konkuk.ac.kr

This supplement provides the Part-2 OLS regression diagnostics referenced in Section 4.4.4 of the main manuscript. The Part-2 OLS specification was estimated on the unweighted analytical subsample with EQ-5D < 1.0 (n = 2,713) using the same control-variable set as the main two-part model. All diagnostic outputs below are reproducible using the KNHANES 2024 data file.

S1. Part-2 OLS Regression Diagnostics

S1.1. Residuals versus fitted values

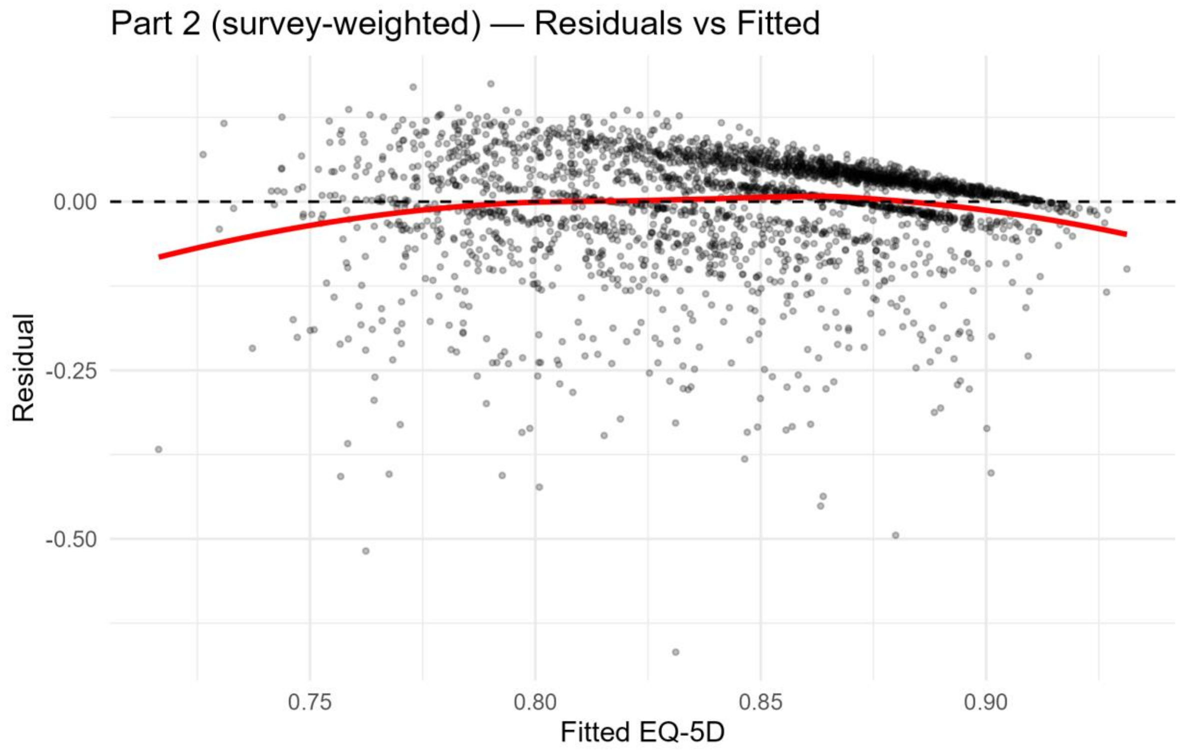

Figure S1. Residuals plotted against fitted EQ-5D values for the Part-2 (survey-weighted) OLS regression on the imperfect-health subsample (n = 2,713). The red line is a LOESS smoother; the dashed horizontal line marks zero.

The residual cloud is asymmetric, with a denser concentration of negative residuals at higher fitted values, reflecting the bounded support of EQ-5D (approaching the upper limit of 1.0). The LOESS fit shows mild downward curvature near the upper boundary, consistent with the heteroscedasticity diagnostic reported in Table S1 below.

S1.2. Normal quantile-quantile plot of residuals

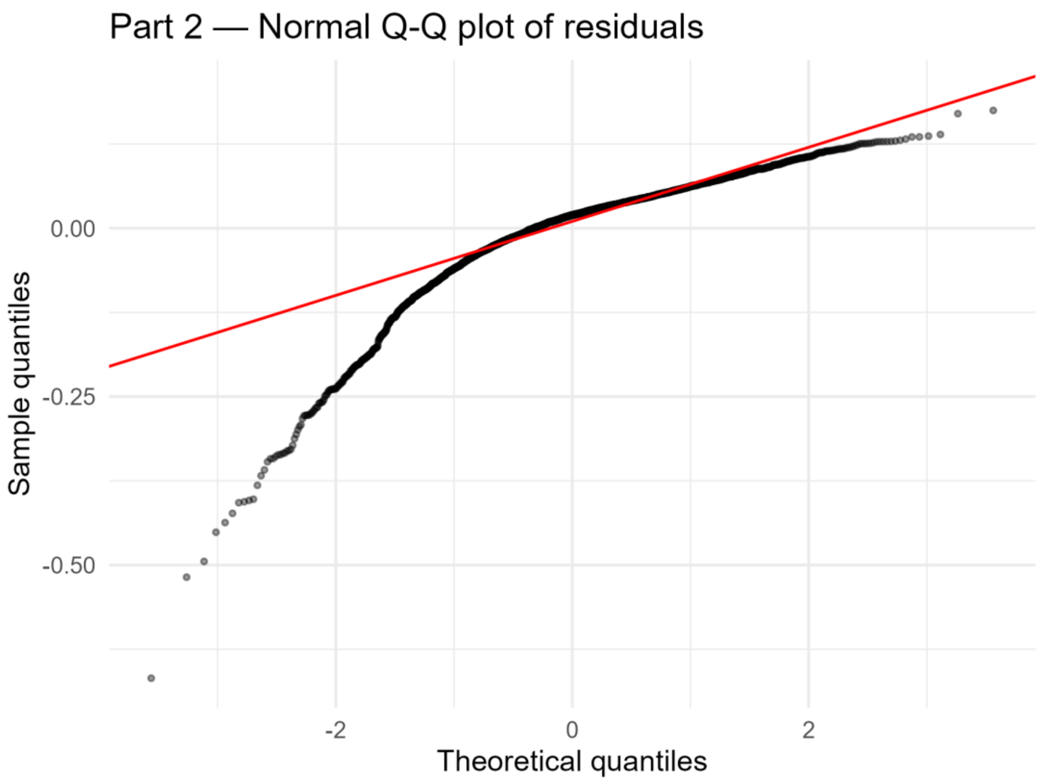

Figure S2. Normal Q-Q plot of the Part-2 OLS residuals. The red line is the theoretical normal quantile reference; deviations from the line indicate departures from normality.

Pronounced left-tail deviations and a flatter right tail are visible, indicating non-normal residuals. The Shapiro–Wilk test in Table S1 formally rejects the normality assumption ($p < .001$). Because survey-weighted OLS does not require residual normality for unbiased point estimation, the practical implication is limited to standard-error inference, which is addressed by the HC3 robust standard errors reported in Table S2.

S1.3. Diagnostic test results

Two formal diagnostic tests were applied to the Part-2 OLS specification: the Shapiro–Wilk test of residual normality and the Breusch–Pagan test of homoscedasticity. The Breusch–Pagan test was run on the unweighted OLS specification, since the standard formulation does not accommodate survey weights; the Shapiro–Wilk test was run on the design-based residuals from the survey-weighted Part-2 model.

Test	Statistic	df	p-value	Conclusion
Shapiro–Wilk (residual normality)	W = 0.85	—	< 0.001	Normality rejected
Breusch–Pagan (homoscedasticity)	BP = 137.0	27	< 0.001	Homoscedasticity rejected

Table S1. Part-2 OLS diagnostic test results (n = 2,713). Both regularity conditions are rejected, motivating the HC3 robust-SE re-analysis reported in Table S2.

S1.4. HC3 robust standard errors versus survey-weighted estimates

Table S2 compares the survey-weighted Part-2 OLS coefficients (used as the main Part-2 specification in §4.2.2 of the manuscript) against an unweighted Part-2 OLS specification with HC3 heteroscedasticity-consistent standard errors. The two specifications differ in (i) whether the KNHANES sampling weights are applied and (ii) the standard-error estimator (model-based vs. HC3 robust). The comparison provides convergent inferential evidence under both the survey-design framework and the formal heteroscedasticity-consistent framework.

Predictor	SW β	SW SE	SW p	HC3 β	HC3 SE	HC3 p
Nutrition label use index	+0.00472	0.00170	0.006	+0.00379	0.00185	0.040
Dietary control	-0.00913	0.00374	0.016	-0.01038	0.00378	0.006
Female (vs. male)	-0.01400	0.00343	<0.0001	-0.01340	0.00398	0.0008
Age (years)	-0.00092	0.00017	<0.0001	-0.00095	0.00017	<0.0001
Education: middle school (vs. elementary or below)	+0.02506	0.00747	0.001	+0.01847	0.00773	0.017
Education: high school (vs. elementary or below)	+0.03785	0.00726	<0.0001	+0.03843	0.00666	<0.0001
Education: college or above (vs. elementary or below)	+0.04564	0.00754	<0.0001	+0.04670	0.00694	<0.0001
Income quintile Q2 (vs. Q1)	+0.01490	0.00530	0.006	+0.01757	0.00586	0.003
Income quintile Q3 (vs. Q1)	+0.01727	0.00545	0.002	+0.01722	0.00582	0.003
Income quintile Q4 (vs. Q1)	+0.01481	0.00542	0.007	+0.01465	0.00584	0.012
Income quintile Q5 (vs. Q1)	+0.01740	0.00636	0.007	+0.01896	0.00578	0.001
Occupation: clerical (vs. manager/professional)	-0.00388	0.00544	0.477	-0.00306	0.00577	0.596
Occupation: service/sales (vs. manager/professional)	-0.00828	0.00537	0.126	-0.00850	0.00585	0.146
Occupation: agriculture (vs. manager/professional)	-0.02518	0.01246	0.045	-0.01872	0.01163	0.108
Occupation: skilled labor (vs. manager/professional)	-0.00334	0.00720	0.643	+0.00007	0.00703	0.992
Occupation: elementary labor (vs. manager/professional)	-0.00089	0.00692	0.898	+0.00354	0.00696	0.612
Occupation: unemployed/inactive (vs. manager/professional)	-0.01940	0.00513	0.0002	-0.02098	0.00511	<0.0001
Marital status: unmarried (vs. married)	-0.01446	0.00555	0.010	-0.01571	0.00591	0.008
Living: couple only (vs. living alone)	+0.00607	0.00495	0.223	+0.00769	0.00451	0.088
Living: couple with children (vs. living alone)	+0.00624	0.00541	0.251	+0.00779	0.00587	0.185
Living: other arrangement (vs. living alone)	-0.00773	0.01491	0.605	-0.00550	0.02223	0.804
Current smoker (vs. non-smoker)	-0.01524	0.00532	0.005	-0.02088	0.00623	0.0008
Drinking frequency (0–6)	+0.00144	0.00095	0.133	+0.00187	0.00114	0.102
Sedentary time (hours/day)	-0.00286	0.00067	<0.0001	-0.00370	0.00068	<0.0001
Walking days (per week)	+0.00011	0.00065	0.865	+0.00026	0.00068	0.698
Average sleep duration (hours/day)	+0.00100	0.00145	0.490	+0.00104	0.00163	0.525
Unmet medical need (yes vs. no)	-0.02689	0.00575	<0.0001	-0.02862	0.00630	<0.0001

Table S2. Survey-weighted (SW) Part-2 OLS coefficients with model-based standard errors versus unweighted Part-2 OLS with HC3 robust standard errors. Coefficients are unstandardized β with EQ-5D as the dependent variable (n = 2,713). p-values are two-sided.

Inferential conclusions converge across the two specifications. The focal nutrition-label-use coefficient retains statistical significance (HC3: β = +0.00379, p = 0.040), albeit with mild attenuation relative to the survey-weighted estimate (β = +0.00472, p = 0.006). The dietary-control, female-gender, education, income, current-smoking, sedentary-time, and unmet-medical-need associations are robust in sign, magnitude, and significance. The agricultural-occupation contrast and the marital-status contrast attenuate slightly under HC3 estimation. No coefficient changes sign between specifications.

S2. STROBE Reporting Checklist

Table S3 below presents the completed Strengthening the Reporting of Observational Studies in Epidemiology (STROBE) checklist for cross-sectional studies, documenting item-by-item compliance with the STROBE 22-item reporting standard. Section/page references point to the corresponding location in the main manuscript.

Item No.	Recommendation	Location in manuscript
Title and abstract		

1 (a)	Indicate the study's design with a commonly used term in the title or the abstract.	Title: 'Two-Part Model Analysis of Nationally Representative Survey Data'. Abstract Methods explicitly identifies KNHANES 2024 as the data source.
1 (b)	Provide in the abstract an informative and balanced summary of what was done and what was found.	Abstract — Background, Methods, Results, Conclusions structure with N, outcome, exposure, modeling approach, and focal coefficients.
Introduction		
2	Explain the scientific background and rationale for the investigation.	§1 (Introduction), paragraphs 1–4.
3	State specific objectives, including any prespecified hypotheses.	§1 final paragraph; §1 new behavioral-theory paragraph (HBM, SCT, health literacy anchoring).
Methods		
4	Present key elements of study design early in the paper.	§3.1 (KNHANES 2024 cross-sectional, stratified multistage cluster design).
5	Describe the setting, locations, and relevant dates, including periods of recruitment, exposure, follow-up, and data collection.	§3.1 (KNHANES 2024, Republic of Korea, household visits + mobile examination centers).
6	Eligibility criteria, sources and methods of selection of participants.	§3.1 (Korean civilian non-institutionalized population aged ≥1; analytical sample restricted to adults 19–80; eligibility/exclusion sequence given).
7	Clearly define all outcomes, exposures, predictors, potential confounders, and effect modifiers.	§3.2.1 (HRQoL outcome via EQ-5D-3L); §3.2.2 (nutrition-label-use index cumulative-stage definition; dietary control); §3.2.3 (full sociodemographic and health-behavior covariate set).
8	For each variable, give sources of data and details of methods of assessment.	§3.2 (KNHANES item codes for every variable, including LK_LB_CO/LK_LB_US/LK_LB_EF for the label items, N_DIET for dietary control, EQ-5D module, and all controls).
9	Describe any efforts to address potential sources of bias.	§3.3 (complex survey-design adjustment); §4.4.3 (multiple imputation for missing data); §4.4.4 (HC3 robust SE for heteroscedasticity); §5.3 (residual-confounding discussion).
10	Explain how the study size was arrived at.	§3.1 (KNHANES 2024 raw N = 6,997; adults 19–80; analytical N = 5,215 after listwise deletion; Part-2 subsample n = 2,713).
11	Explain how quantitative variables were handled in the analyses.	§3.2 (continuous predictors entered linearly; categorical predictors as factor dummies); §3.3 (two-part model rationale for ceiling effect); §4.4.2 (ordinal sensitivity analysis confirming linearity of the index).
12 (a)	Describe all statistical methods, including those used to control for confounding.	§3.3 (survey-weighted logistic for Part 1; survey-weighted OLS for Part 2; comprehensive control set).
12 (b)	Describe any methods used to examine subgroups and interactions.	§4.4.5 (lb_index × diet_ctrl interaction model; stratified estimates by diet_ctrl).
12 (c)	Explain how missing data were addressed.	§3.1 (listwise-deletion main analysis with included/excluded comparison); §4.4.3 (multiple imputation re-analysis with m = 10, MICE).
12 (d)	If applicable, describe analytical methods taking account of sampling strategy.	§3.3 (kstrata, psu, wt_tot complex-survey design; nested clusters; design-based standard errors).
12 (e)	Describe any sensitivity analyses.	§4.3 (full-sample OLS); §4.4.1 (component decomposition: joint and single-component); §4.4.2 (ordinal index with polynomial trend); §4.4.3 (multiple imputation); §4.4.4 (HC3 robust SE; see Supplementary Tables S1–S2 and Figures S1–S2); §4.4.5 (interaction and stratification).
Results		
13 (a)	Report numbers of individuals at each stage of study.	§3.1 (raw 6,997 → adults 19–80 → analytical 5,215; Part-2 subsample 2,713).
13 (b)	Give reasons for non-participation at each stage.	§3.1 (listwise-deletion exclusion summary; primary causes were item non-response on label use, dietary control, and sedentary time).

13 (c)	Consider use of a flow diagram.	Inclusion/exclusion sequence stated narratively in §3.1; CONSORT-style flow diagram available on editorial request (R2-5).
14 (a)	Give characteristics of study participants and information on exposures and potential confounders.	§4.1 with Table 1 (weighted sample characteristics) and Table 2 (weighted descriptive statistics for continuous variables).
14 (b)	Indicate number of participants with missing data for each variable of interest.	§3.1 (overall 25.5% excluded); §4.4.3 (variable-level missingness summary in MI re-analysis: e.g., sedentary 478, walk_days 422, occupation 433, label-index 0 within MICE-eligible cases).
15	Report numbers of outcome events or summary measures.	§4.1 (weighted EQ-5D mean = 0.928; perfect-health share = 48.0%); §4.2.1 (Part-1 OR table); §4.2.2 (Part-2 β table); Table 6 (sensitivity analyses).
16 (a)	Give unadjusted estimates and, if applicable, confounder-adjusted estimates and their precision.	§4.1 (Pearson correlations as unadjusted associations); §4.2.1 / §4.2.2 (adjusted Part-1 OR and Part-2 β with 95% CIs).
16 (b)	Report category boundaries when continuous variables were categorized.	§3.2.3 (income quintile boundaries via equivalized household income; occupational categories via Korean Standard Classification of Occupations); drinking-frequency 7-point scale defined in Table 2 note.
16 (c)	If relevant, consider translating estimates of relative risk into absolute risk for a meaningful time period.	Not applicable — outcome is a continuous utility index, not a relative-risk measure; magnitude interpretation against MID benchmarks is provided in §5.1.1.
17	Report other analyses done — e.g., subgroups, interactions, sensitivity.	§4.3 (full-sample OLS robustness); §4.4.1–§4.4.5 (five sensitivity analyses); Table 6; Figures 6–8; Supplementary Figures S1–S2 and Tables S1–S2.
Discussion		
18	Summarise key results with reference to study objectives.	§5.1 opening (associative-framing paragraph); §5.1.1 (effect-size discussion); §5.1.2 (differential pattern across the two-part model); §5.1.3 (dietary-control paradox); §6 (Conclusions).
19	Discuss limitations of the study, taking into account sources of potential bias or imprecision.	§5.3 (six explicit limitations: cross-sectional design; self-report; absence of mental-health and chronic-disease indicators; listwise-deletion selection; heterogeneity of dietary-control item; residual confounding from literacy and food-environment factors).
20	Give a cautious overall interpretation considering objectives, limitations, multiplicity of analyses, results from similar studies, and other relevant evidence.	§5.1 framing paragraph (associative, not causal); §5.1.1–§5.1.4; comparisons with Cheng et al. [20] and prior KNHANES analyses [21,22].
21	Discuss the generalisability (external validity) of the study results.	§5.2 (Practical Implications, including comparisons to Chile/AU-NZ/France FOP regimes); §5.3 (caveat that Korean back-of-pack regime may attenuate or distort comparison with FOP-system populations).
Other information		
22	Give the source of funding and the role of the funders for the present study and, if applicable, for the original study on which the present article is based.	§ Acknowledgments and Funding statements at the end of the manuscript (KU research Program; no external funding for the present analysis).

Table S3. STROBE statement — checklist of items that should be included in reports of cross-sectional studies. Section headers (Title and abstract, Introduction, Methods, Results, Discussion, Other information) are listed without item numbers; the 22 numbered checklist items follow each header.

Reference: von Elm E, Altman DG, Egger M, Pocock SJ, Gøtzsche PC, Vandenbroucke JP; STROBE Initiative. The Strengthening the Reporting of Observational Studies in Epidemiology (STROBE) statement: guidelines for reporting observational studies. Annals of Internal Medicine 2007;147(8):573–577.
